# An Ultra-Rare Mixed Phenotype with Combined AP-4 and ERF Mutations: The First Report in a Pediatric Patient and a Literature Review

**DOI:** 10.3390/genes15040436

**Published:** 2024-03-29

**Authors:** Alessandro Orsini, Andrea Santangelo, Alessandra Carmignani, Anna Camporeale, Francesco Massart, Nina Tyutyusheva, Diego Giampietro Peroni, Thomas Foiadelli, Alessandro Ferretti, Benedetta Toschi, Silvia Romano, Alice Bonuccelli

**Affiliations:** 1 Pediatric Neurology, Pediatric Department, AOUP Santa Chiara Hospital, 56100 Pisa, Italy; aorsini.md@gmail.com (A.O.); al.bonuccelli@gmail.com (A.B.); 2 Department of Neurosciences, Rehabilitation, Ophthalmology, Genetics, Maternal and Child Health, University of Genoa, 16126 Genoa, Italy; 3 Pediatric Department, AOUP Santa Chiara Hospital, 56100 Pisa, Italy; alessandra.carmignani1@gmail.com (A.C.); annacamporeale22@gmail.com (A.C.); diego.peroni@unipi.it (D.G.P.); 4 Pediatric Endocrinology, Pediatric Department, AOUP Santa Chiara Hospital, 56100 Pisa, Italy; massart@med.unipi.it (F.M.); nina.tyutyusheva@gmail.com (N.T.); 5 Clinica Pediatrica, Fondazione IRCCS Policlinico San Matteo, 27100 Pavia, Italy; t.foiadelli@smatteo.pv.it; 6 Pediatrics Unit, Neuroscience, Mental Health and Sense Organs (NESMOS) Department, Faculty of Medicine and Psychology, Sapienza University of Rome, 00185 Rome, Italy; alessandro.ferretti@uniroma1.it; 7 Division of Medical Genetics, Department of Medical and Oncological Area, University-Hospital, 56126 Pisa, Italy; b.toschi@ao-pisa.toscana.it (B.T.); silvia.romano@ao-pisa.toscana.it (S.R.)

**Keywords:** AP-4, ERF, epilepsy, AP-4 deficiency syndrome, ERF-related craniosynostosis, hereditary spastic paraparesis

## Abstract

The adaptor protein 4 (AP-4) constitutes a conserved hetero-tetrameric complex within the family of adaptor protein (AP) complex, crucial for the signal-mediated trafficking of integral membrane proteins. Mutations affecting all subunits of the AP-4 complex have been linked to autosomal-recessive cerebral palsy and a complex hereditary spastic paraparesis (HSP) phenotype. Our report details the case of a 14-year-old boy born to consanguineous parents, presenting psychomotor delay, severe intellectual disability, microcephaly, and trigonocephaly. Despite a history of febrile seizures, subsequent years were devoid of seizures, with normal EEG. Exome sequencing revealed pathogenic variants in both the *AP4B1* and *ERF* genes. Significantly, the patient exhibited features associated with *AP4B1* mutations, including distinctive traits such as cranial malformations. The *ERF* gene variant, linked to craniosynostosis, likely contributes to the observed trigonocephaly. This case represents the initial documentation of a concurrent mutation in the *AP4B1* and *ERF* genes, underscoring the critical role of exome analysis in unraveling complex phenotypes. Understanding these complex genotypes offers valuable insights into broader syndromic conditions, facilitating comprehensive patient management.

## 1. Introduction

The adaptor protein 4 (AP-4) constitutes a conserved hetero-tetrameric complex within the family of adaptor protein (AP) complex, which are pivotal in the signal-mediated trafficking of integral membrane proteins [1]. AP-4 consists of two large subunits, namely β (AP-4B1) and epsilon (AP4E1), a medium subunit (AP4M1), and a small subunit (AP4S1) [2].

AP4B1 encodes the β1 subunit of the AP-4 complex, which, alongside AP-1, AP-2, AP-3, and AP-5, forms part of the adaptor protein family. AP complexes 1–5 undergo transient transportation into membranes, where they act as coat proteins, facilitating cargo selection and vesicle shaping. These proteins exhibit ubiquitous expression across human tissues and play a fundamental role in vesicle trafficking.

The functions of AP-4 are manifold, with studies demonstrating its distinctive involvement in the trafficking of α-amino-3-hydroxy-5-methyl-4-isoxazolepropionic acid (AMPA) receptors for glutamate in neurons [3]. AMPA receptors are highly expressed in migrating interneurons and immature oligodendrocytes during the onset of myelination [4]. Mutations affecting all subunits of the AP-4 complex have been associated with autosomal-recessive cerebral palsy or the complex hereditary spastic paraparesis (HSP) phenotype.

The *ERF* gene, also recognized as the ETS2 repressor factor gene, is responsible for encoding a transcriptional repressor crucial in the development and differentiation of various tissues, including bone and cartilage. Pathogenic alterations within the *ERF* gene have been linked to an infrequent autosomal dominant disorder identified as ERF-related craniosynostosis [5,6,7].

The simultaneous occurrence of different mutations leads to the development of complex phenotypes sharing combined characteristics of both syndromes.

Illustrating cases of ultra-rare individuals with variants in both *AP-4* and *ERF* genes not only enhances our comprehension of rare congenital syndromes but also has significant implications for clinical management. Delving into the molecular intricacies of these conditions opens avenues for potential targeted therapies that address the underlying genetic mechanisms. Furthermore, such descriptions play a pivotal role in anticipating potential complications or associated conditions linked to the identified genetic variants. This insight could be pivotal for prognostic considerations, early diagnosis, and guiding genetic counseling and family planning decisions.

## 2. Materials and Methods

We conducted a systematic literature review utilizing multiple electronic databases, including PubMed/Medline, Embase, and Web of Science, with the objective of comprehensively identifying and analyzing original research papers, meta-analyses, clinical trials, and reviews concerning mutations in AP-4 subunits. Our focus was on publications in English within the last 15 years, from January 2008 to December 2023. To ensure a thorough search, two authors independently undertook a literature review, identifying studies that provided insights into the clinical phenotypes associated with AP-4 variants.

Various study designs were considered, including systematic and narrative reviews, preclinical and clinical trials, as well as observational studies, all derived from human data. Additionally, our exploration encompassed an understanding of pathogenetic mechanisms. The search strategy employed specific keywords related to AP-4 mutations and potential variants in other genes, either used individually or in combination to retrieve relevant literature sources. Primary keywords included “adaptor protein 4”, “AP-4”, “AP-4 deficiency syndrome”, and “hereditary spastic paraparesis”. Special attention was given to studies conducted on infants, children, and adolescents to address the specific implications of AP-4 mutations in these age groups.

Data from selected studies were meticulously extracted based on their relevance to the topic and subsequently analyzed to provide a comprehensive overview of the current understanding of the various phenotypes associated with these AP-4 variants

DNA sequencing of the proband and parents was conducted using a Paired-End 150 bp protocol on a NexiSeq 500 sequencer (Illumina, San Diego, CA, USA). Prior to sequencing, selective enrichment of coding regions was performed using a SureSelectXT2 Clinical Research Exome (Agilent Technologies, Santa Clara, CA, USA) or a Twist Human Core Exome Kit (Twist Biosciences, South San Francisco, CA, USA). Quality parameters required for analysis included average coverage exceeding 60 reads per nucleotide, >95% of target bases covered at >20×, and >92% of target bases covered at >30×.

## 3. Case Report

We present the case of a 14-year-old male exhibiting severe intellectual disability, psychomotor developmental delay, microcephaly, and trigonocephaly, characterized by a prominent metopic ridge, along with a history of febrile seizures. He was born to consanguineous parents (first cousins) after a full-term pregnancy, delivered spontaneously at 40 weeks gestational age, with a birth weight of 4800 g. Prenatal ultrasounds did not reveal any abnormalities.

At the age of six months, he was hospitalized following febrile seizures triggered by the second dose of the hexavalent vaccine (comprising diphtheritis, tetanus, pertussis, poliovirus, hepatitis B virus, and H. influenzae B). Subsequently, there were no recurrences of febrile seizures in subsequent years, and EEG results remained normal.

Upon our first examination of the patient at 9 years of age, the patient exhibited severe intellectual disability and lacked independent walking ability. Additionally, he presented with severe speech impairment (expressive language limited to vocalizations and gestures), though he demonstrated the ability to understand and execute simple commands. Physical examination revealed microcephaly and trigonocephaly, with a low anterior hairline, alteration of the auricles with folded helices, hypertrichosis of the back and forehead, clinodactyly affecting the second and third toes, and bradydactyly in the hands.

**Figure 1:**
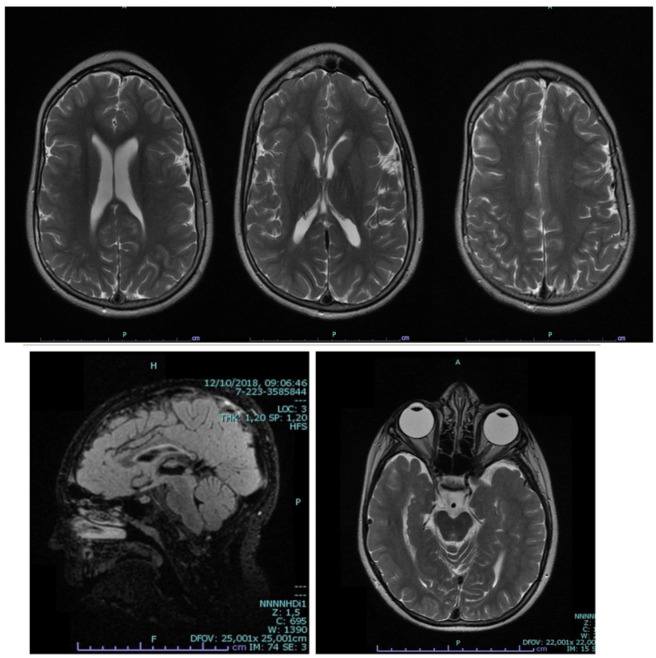
MRI of the reported patient showing the shape of the head, thinning of the corpus callosum, and white matter hypotrophy.

The neurological examination revealed hyper-reflexia, hypertonia of both upper and lower limbs, and notable joint stiffness, particularly in the lower limb joints. A brain MRI, conducted at the age of 10, unveiled thinning of the corpus callosum, along with subependymal and periventricular white matter hypotrophy. Additionally, ectopic neurohypophysis was observed, suggesting a potential link to the endocrine abnormalities present in our patient (Figure 1).

Various genetic analyses, including karyotyping, CGH-array, multiplex ligation-dependent probe amplification (MLPA) analysis, and screening for fragile X syndrome, all yielded negative results. Consequently, exome sequencing (ES) was pursued, revealing the presence of the following variants:

- A homozygous c.1793-2A>G variant in the *AP4B1* gene (c.1793-2A>G), which was detected in heterozygosity in the mother.
- A heterozygous c.1201_1202delAA variant in the *ERF* gene (p.Lys401GlufsTer10), which was absent in the mother.

Both variants were classified as pathogenic according to the American College of Medical Genetics and Genomics and the Association for Molecular Pathology (ACMG-AMP) variant interpretation guidelines. Notably, the identified deletion in *ERF* has been described as pathogenic, since it is predicted to result in a truncated protein, whereas the *AP4B1* variant has never been reported in the literature. Unfortunately, segregation analysis was hindered by the previous death of the father, due to causes unrelated to our patient’s disease, therefore rendering it unfeasible to ascertain the inheritance pattern of these alterations.

## 4. Discussion and Literature Review

Individuals carrying pathogenic variants in the *AP4B1* gene usually present with clinical heterogeneous features, making diagnosis challenging.

Pathogenic variants in all subunits of the AP-4 complex have been associated with a rare autosomal-recessive disorder known as hereditary spastic paraparesis, also called AP-4 deficiency syndrome, an extremely rare disease observed in about 115 patients worldwide [8]. Onset generally occurs within the first year of life, with hypotonia, microcephaly, and developmental delays, evolving into progressive lower-extremity weakness and spasticity with pyramidal features. Many affected children become non-ambulatory, relying on mobility aids, as spasticity extends to the upper extremities, resulting in spastic tetraplegia [9].

Complications include dysphagia, contractures due to progressive spasticity, foot deformities, and disruptions in bladder and bowel function. Microcephaly is prevalent, often falling within the −2 SD to −3 SD range, and developmental delays universally impact motor milestones and speech. Intellectual disability, typically moderate to severe, is common in older patients [9,10].

Seizures occur in approximately 50% of individuals with AP-4-associated HSP, manifesting in the first two years of life. Seizure types include focal-onset and primary generalized seizures. While seizures tend to become less frequent with age, stereotypic laughter, potentially indicating a pseudobulbar effect, remains a characteristic feature in some cases [10]. This peculiar finding adds a specific dimension to the diagnostic criteria, and it has been suggested that it could support the differentiation of AP-4-associated HSP from other conditions with overlapping symptoms [10].

Less frequent clinical manifestations encompass short stature, non-specific dysmorphic facial features, optic nerve atrophy, dystonia, and ataxia. Notably, uncomplicated hereditary spastic paraplegia has not been documented in AP-4 deficiency cases. Common brain anomalies that are observed in this clinical context include thinning of the corpus callosum, observed in a substantial majority of cases (90%), predominantly involving the splenium. Nonetheless, non-specific T2 signal changes in the supratentorial white matter are common, primarily concentrated in the periventricular area. Ventriculomegaly is also prevalent (65%), often manifesting as asymmetric colpocephaly, likely stemming from the loss of periventricular white matter volume. Global cerebral atrophy can be detected in up to 37% of patients, appearing even in toddlers and young children but becoming more apparent in older patients with advanced disease progression. While cerebellar atrophy is generally infrequent, it is evident in certain patients, particularly those with advanced disease [11]. Less common imaging findings include symmetric iron deposition in the globus pallidus and bilateral symmetric polymicrogyria [12,13]. In particular, ventriculomegaly, white matter loss, and thinning of the corpus callosum have been proposed as key features of AP-4-associated HSP [14].

Notably, uncomplicated hereditary spastic paraplegia has never been reported in AP-4 deficiency cases, and prognosis details remain limited. The oldest reported individuals are young adults, highlighting the need for further natural history data.

To date, several types of mutations have been identified in the *AP4B1* gene among individuals with AP-4 deficiency syndrome. Most of these mutations predictably result in a loss of AP-4 function, impairing the transport of proteins and lipids within cells [5]. Some of the variants identified in the *AP4B1* gene are missense mutations, altering a single amino acid in the AP4B1 protein [15], while others are nonsense mutations or frameshift mutations disrupting normal protein production [4].

As shown in Table 1**,** seizures and febrile seizures are reported in the literature in the majority of patients with AP-4 variants, with some authors reporting stereotypic laughter and shy behavior [2,4,15,16,17,18,19,20]. Recognizable clinical features associated with mutations in AP-4 complex subunits have led to the term “AP-4 deficiency syndrome”. Besides *AP4B1*, mutations on the other three subunits can also cause autosomal-recessive HSPs. Interestingly, our patient presented many of the characteristics linked with *AP4B1* mutations, whereas the association with other features like trigonocephaly, craniosynostosis, hypertrichosis, and clinodactyly has never been reported.

Several mutations in the *ERF* gene have been identified in individuals with craniosynostosis. In fact, the overall prevalence of *ERF* mutations in patients with syndromic craniosynostosis stands at around 2% and at 0.7% in clinically non-syndromic craniosynostosis [21].

As initially demonstrated by Twigg et al., reduced expression of *ERF*, encoding an inhibitory ETS transcription factor, directly modulated by ERK1/2, leads to the development of complex craniosynostosis in both humans and mice. Interestingly, the authors highlighted that such a clinical disorder could manifest with multiple suture synostosis, craniofacial dysmorphism, the presence of Chiari malformation, and language development delays [7].

Mutations occurring in the *ERF* gene are usually missense or frameshift [21,22]. Some of these variants affect critical functional domains of the protein, such as the DNA-binding domain or the repression domain, and are predicted to impair the ability of ERF to properly regulate gene expression.

Some individuals with ERF-related craniosynostosis may also have other physical abnormalities, such as hypertelorism, pinna abnormalities, exophthalmos, and abnormalities of the ears or teeth. Mild to moderate intellectual disability has also been reported in some affected individuals. Notably, the variant identified in our patient is reported in the reference databases and in the literature in association with the related ERF craniosynostosis. We presume that this alteration might be responsible for some of the characteristics of the patient, in particular *ERF*-related craniosynostosis.

Our report identifies a novel pathogenic variant in the *AP4B1* gene from a patient exhibiting clinical features of hereditary spastic paraplegias, intellectual disability, psychomotor development delay, febrile seizures, and thinning of the corpus callosum. None of these clinical features are specific or distinguish the involvement of different AP-4 subunits. However, the constellation of autosomal-recessive spastic tetraplegia, severe intellectual disability, stereotypical laughter, limited or absent speech, microcephaly, as well as facial and cranial MRI features, should prompt screening for homozygous mutations in any of the four subunits leading to AP-4 deficiency syndrome. Moreover, our patient presented a variant of the *ERF* gene as well, which has been proven to be responsible for craniosynostosis and other malformations. Interestingly, it has been reported that *ERF*-related craniosynostoses appear to occur later than other craniosynostosis syndromes (median age at 42 months) [6], whereas our patient showed trigonocephaly at birth.

The patient displayed a phenotype involving characteristics associated with mutations in both the *AP4B1* and *ERF* genes. To our knowledge, this case study describes the first patient in the literature with a combined mutation in the *AP4B1* and *ERF* genes. The case underscores the importance of exome analysis in patients with a complex phenotype, as broad-spectrum genetic examinations could reveal pathogenic mutations in different genes associated with various syndromic conditions.

## Figures and Tables

**Table 1:** Main characteristics of patients with AP-4 subunit mutations in the literature.

	Present Study	Moreno De Luca et al. (2011) [15]	Ruan et al. (2020) [16]		Accogli et al. (2018) [17]		Tuysuz et al. (2014) [2]			Abou Jamra et al. (2011) [4]			Lamichhane (2013) [18]	Tessa et al. (2016) [19]		
AP-4 subunit mutation	B1	E1	B1		B1	B1	M1	M1	B1	B1	S1	E1	B1	S1		
Variant	c.1793-2A>G	Deletion 15q21.2 222 kb (consanguineous family)	c.1207C > T/c.52_53delAC		c.991C>T, p.Q331X	c.991C>T, p.Q331X	c.1012C>T	c.952C>T	c.869delC	c.487_488insTAT, p.Glu163_Ser739 delinsVal	c.124C>T, p.Arg42*	c.542þ1_542þ4delGTAA, r.421_542del, p.Glu181Glyfs*20	c.311delC, c.577G>A	c.138+3_6delAAGT	c.43C>T p.R15*	c.49dupT p.S17Fƒs*2
Other genes involved	ERF gene heterozygosity	*SPPL2A* (signal peptide peptidase-like-2A) has a role in immune regulation	No		No	No	No	M6PR heterozygosity	No	No			No	No		
Sex, Age at evaluation	M; 14 y	F; 23y	M; 22 y	M; 9 y	M; 16 y	M; 11 y	2 F; 17 y–11 y	M; 10 y	2 F; 12.5 y–10.5 y	2 F, 1 M (siblings); 23 y, 15 y, 11 y.	2 M, 1 F (siblings); 22 y, 20 y, 18 y.	1 F, 1 M; 11 y, 6 y,	M; 4 y	F; 5 y F; 2 y (siblings)	M; 15 y	F; 13 y
Intellectual disability	Moderate	Mild	Mild	Mild	Moderate	Mild	Moderate			Severe			Moderate	Mild (2/2)	Mild	Moderate
Spasticity	Mild	Mild	Mild	Mild	Mild	Mild	2/2			3/3	2/3	2/2	Mild	1/2	Mild	Mild
Speech disorder	Mild	Mild	Mild	-	Mild	Mild	Moderate			2/3	3/3	2/2	Mild	Mild	Mild	Mild
Seizures	FS	+	+ (onset at 15 y)	FS	GTCS	GTCS	+			-			GMS	FS (1/2)	-	FS and FoS
Infantile hypotonia	-	Mild	Mild	-	Mild	Mild	N/A	N/A	Mild	3/3			Mild	N/A		
Hyper-reflexia	Mild	Mild	Mild	Mild	Mild	Mild	Moderate			3/3	2/2, 1 N/A	N/A		N/A	Mild	
Walking (years)	-	N/A	Walk with support	2 y (unstable walk)	-	-	3–5 y			2.5/2.5/2.5			Walking with difficulty	Inability to walk unassisted	3.5	2.5
Short stature	+ (GH deficiency)	N/A	N/A	N/A	-4 SD	N/A	N/A			3/3			N/A	N/A		
Foot deformity	+	N/A	N/A	+	+	N/A	1/2			N/A			N/A	N/A		
Cranial abnormalities	Microcephaly and metopic craniosynostosis	Microcephaly	Microcephaly	-			Microcephaly			Microcephaly			N/A	Microcephaly		
Stereotypic laughter	+	+	+	N/A	-	-	+			3/3	3/3	1/2		N/A		
Facial features		Bitemporal narrowing, pointed chin, down-slanting palpebral fissures, and long nose with a wide nasal ridge	Similar to another patient	N/A	Bitemporal narrowing, thick eyebrows, broad nasal ridge, thick nostrils, and short philtrum	Broad nasal ridge, short philtrum, and bulbous nose	Facial hypotonia, broad nasal ridge, short philtrum, wide mouth, and high palate			Microcephaly, high palate, and wide nasal bridge			N/A	N/A	Mild facial dysmorphisms	Mild facial dysmorphisms
EEG features	Normal	Generalized theta slowing of the background, consistent with diffuse cerebral dysfunction		Normal			Normal			N/A				N/A		
Brain MRI	Thin corpus callosum, white matter hypotrophy, and ectopic neurohypophysis	Ventriculomegaly, cerebellar atrophy, reduced hippocampal volume, and diffuse white matter loss more pronounced in the frontal region of the corpus callosum	Ventriculomegaly with slightly prominent cisterns	Dilated supratentorial ventricle and thin corpus callosum	Ventriculomegaly, thin corpus callosum, and paucity of white matter	Ventriculomegaly and diffuse cortical and subcortical atrophy	Ventriculomegaly, thin splenium of the corpus callosum, white matter loss, and hippocampal globoid formation			N/A			No structural abnormalities	Thinning of the body of the splenium of the corpus callosum	Thinning of the body of the splenium of the corpus callosum	Thinning of the splenium of the corpus callosum
Other features	Hypertrichosis, clinodactyly	N/A		Lumbar lordosis	Left Horner syndrome, with ptosis and myosis, cataracts, and optic nerve atrophy	Syndactyly of the second and third toes bilaterally	N/A			N/A			N/A.	N/A		

Legend: M: male; F: female; FS: febrile seizures; GTCS: generalized tonic–clonic seizures; FoS: focal seizures; GMS: generalized myoclonic seizures; SD: standard deviation; N/A: not available; +: present, -: absent.

## Data Availability

Data are contained within the article.
